# Kidney-targeted nanoplatforms: Strategies and applications

**DOI:** 10.7150/thno.126217

**Published:** 2026-01-01

**Authors:** Yucen Deng, Ziyu Liu, Xinyuan Zhu, Youfu Wang, Xuesong Feng, Jinghui Yang

**Affiliations:** 1 Department of Organ Transplantation, Shanghai Changzheng Hospital, Naval Medical University, Shanghai 200003, China.; 2 School of Chemistry and Chemical Engineering, State Key Laboratory of Polyolefins and Catalysis, Research Institute of Polymer Materials, Shanghai Jiao Tong University, Shanghai 200240, China.; 3 School of Pharmacy, China Medical University, Shenyang 110122, China.; 4 Department of Pharmacy, Chongqing Hospital, Union Hospital, Tongji Medical College, Huazhong University of Science and Technology, Chongqing 401121, China.; 5 Department of Nephrology, Shanghai Changzheng Hospital, Naval Medical University, Shanghai 200003, China.

**Keywords:** kidney-targeted, nanoplatforms, nanodelivery, structure-function relationships

## Abstract

Renal diseases remain a major global health burden, with an estimated 850 million individuals affected by chronic kidney disease, acute kidney injury, glomerulonephritis, and diabetic nephropathy. These multifactorial diseases collectively account for substantial morbidity and mortality burdens. This grim trajectory demands urgent development of drugs that are capable of simultaneously enhancing renal efficacy while circumventing systemic toxicity. In response to this challenge, engineered nanoplatforms designed specifically for the treatment of kidney diseases have emerged as a promising solution. These nanoplatforms offer the unique ability to deliver targeted therapeutics directly to specific regions of the kidney, thereby improving drug efficacy while reducing off-target effects. Unlike the well-established oncological applications of nanomedicine, renal-specific formulations remain in their developmental nascency. Nevertheless, accumulating preclinical evidence indicates that nanotherapeutics hold significant promise for improving the clinical management of kidney diseases through targeted and mechanism-based interventions. The nephrotropic mechanisms and structural determinants of renal nanoplatforms fundamentally diverge from those of conventional nanotherapeutics. Therefore, a thorough understanding of the principles governing renal targeting is essential for designing nanomedicines that achieve precise kidney-specific delivery while ensuring biosafety. In this review, we summarize the current understanding of structure-function relationships that govern the targeting efficiency and biodistribution of nanoparticles in the kidney, with a focus on passive targeting mechanisms driven by key physicochemical parameters, such as particle size, surface charge, shape, and density, as well as active targeting strategies based on specific receptor-ligand interactions.

## 1. Introduction

The human kidney, a retroperitoneal organ with a distinctive biconcave morphology 1-4, serves as a master regulator of systemic homeostasis 5-8. Beyond its canonical role in urinary solute excretion and fluid-electrolyte balance 9, this organ orchestrates multifaceted physiological processes: modulating circulatory dynamics through renin-angiotensin-aldosterone system activation 10, 11, maintaining acid-base equilibrium *via* bidirectional proton-bicarbonate transport 12, and governing endocrine functions *via* erythropoietin synthesis and vitamin D hydroxylation 13-15. Such integrative mechanisms not only sustain calcium-phosphate metabolism and erythroid progenitor differentiation but also mediate the catabolism of circulating hormones, thereby preserving the stability of the internal milieu 16. Consequently, any impairment or loss of kidney function may give rise to a series of severe health complications 17-19, such as nephritis, acute kidney injury (AKI), and renal fibrosis 20-24.

Contemporary pharmacotherapeutic paradigms face dual challenges in achieving renal efficacy. While optimal drug biodistribution necessitates sufficient parenchymal accumulation, pathologically altered renal architecture—compromised glomerular filtration barriers (GFB) 25-28, dysregulated tubular transport 29, 30, or cystic microenvironment remodeling—frequently disrupts conventional pharmacokinetic profiles. Compounding this complexity, intracellular drug bioavailability is governed by a tripartite interplay of membrane transporter dynamics, metabolic enzyme activity, and tissue partitioning kinetics 31-33. These biological barriers render simplistic dose-escalation strategies pharmacodynamically suboptimal, amplifying risks of off-target cytotoxicity and multidrug interactions that may paradoxically exacerbate nephrotoxicity 34, 35. Such limitations underscore an urgent imperative: the rational design of spatially precise, cell-selective delivery platforms capable of navigating renal anatomical and molecular landscapes 36, 37.

Emerging nanotherapeutics offer transformative solutions to these challenges 38-46. Engineered nanoplatforms enable programmable interactions with renal cells through controlled surface chemistry and ligand functionalization 47-49. Their ability to carry diverse payloads—small molecules, biologics, nucleic acids, and imaging probes—supports combined therapeutic and diagnostic applications. Preclinical studies show improved pharmacokinetics by evading reticuloendothelial clearance and enabling stimuli-responsive drug release in diseased renal tissues 50-56. This review outlines two strategies: 1) Passive targeting based on intrinsic renal pathophysiology; 2) Active targeting using ligand-receptor recognition across renal cells. By integrating nanotechnology and nephrology perspectives, this work explores potential design principles for next-generation renal nanomedicines. We hope this review may help facilitate cross-disciplinary efforts toward advancing clinically viable renal-targeted nanotherapeutics.

## 2. Passive renal targeted delivery strategy

**Figure 1:**
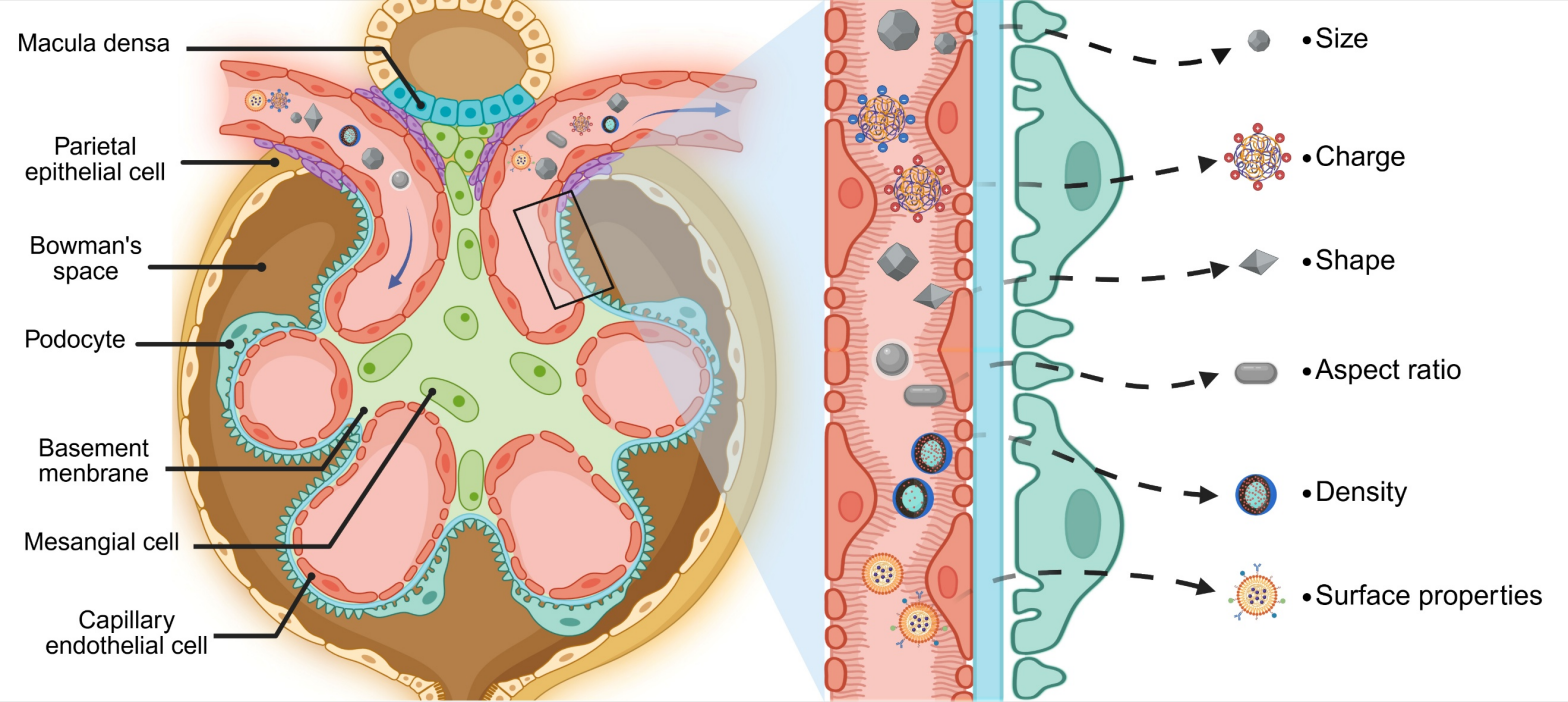
** Glomerular architecture and filtration selectivity.** The glomerular filtration barrier establishes a size- and charge-selective pathway for nanoparticles traversing from glomerular capillaries to Bowman's space. This tripartite system requires sequential penetration through: (1) A fenestrated endothelial layer coated with glycocalyx proteoglycans, where subendothelial pores of 60-80 nm diameter permit initial size exclusion. (2) The GBM, a charge-selective matrix rich in collagen IV and laminin, whose 10 nm interstitial channels enforce secondary electrostatic filtration *via* heparin sulfate-mediated anionic repulsion. (3) Podocyte-derived slit diaphragms spanning 12 nm gaps between interdigitating foot processes, stabilized by nephrin-podocin complexes that regulate final molecular sieving. Collective interplay between these architectonic features—dynamic pore geometries, electrostatic barriers, and protein-regulated permselectivity—dictates the renal targeting profiles of nanoplatforms through synergistic biophysical discrimination mechanisms. Created with Biorender.com.

Current biomedical approaches for renal targeting primarily employ two distinct mechanisms: active targeting and passive targeting. Active targeting utilizes ligand-receptor interactions or antibody-antigen recognition to achieve cell-specific drug delivery through molecular recognition of membrane-bound biomarkers 57-60. In contrast, passive targeting exploits inherent physiological gradients and anatomical barriers to mediate differential drug distribution (**Figure 1**).

### 2.1 Size

The unique structure of glomerular capillaries, characterized by open windows with diameters ranging from 60-80 nm, along with the glomerular basement membrane (GBM) exhibiting an average pore size of 10 nm and cleft septa measuring 12-22 nm in diameter at pedicle protrusions of podocytes, presents an inherent challenge for drug molecules attempting to pass through this barrier 61-63. Nanoparticle interaction with this tripartite barrier exhibits strict size dependency: particles exceeding 5.5 nm HD demonstrate prolonged circulation but enhanced reticuloendothelial system uptake, while those below this threshold undergo renal clearance 64-66. Intriguingly, NPs between 10-150 nm display unique glomerular trafficking behaviors, traversing endothelial fenestrations yet encountering GBM-mediated retention 67-69. This size window enables selective accumulation in glomerular subdomains, including tethered membranes and mesangial regions, offering therapeutic potential for inflammatory and fibrotic nephropathies 70-72. Experimental validation of size-dependent targeting was demonstrated by Hao Wei *et al*. through polyvinylpyrrolidone-curcumin NPs (PCurNPs) of varying diameters 73. Comparative analysis revealed 89Zr-PCurNP M10 (5-8 nm) achieved 1.7- to 1.8-fold greater renal accumulation than larger variants (20-50 nm), correlating with improved histopathological outcomes (reduced serum creatinine, attenuated tubular injury scores). These findings confirm that optimal HD (< 10 nm) maximizes nephroprotective efficacy through enhanced glomerular permeation.

### 2.2 Surface charge

Surface charge is another important fact when considering the passive targeting efficacy and renal accumulation. Given that the glomerular filtration window is markedly negatively charged, it exerts a pronounced repulsive force on negatively charged particles, preventing them from passing through 74. In the contrary, it has been observed that nanoparticles with a positive potential are more readily filtered and tend to exhibit higher renal accumulation, leading to enhanced efficacy and prolonged half-lives for payloads 75, 76. However, this must be balanced against potential drawbacks such as non-specific protein adsorption that may result from elevated levels of positive charge 77-79. Beyond simply being positively or negatively charged, the specific magnitude of charge also plays a crucial role in determining the fate of particles within the glomerular filtration window. To explore the influence of surface charge on NPs in the kidney, Roberts' team synthesized ultrasmall (3.7 nm) water-dispersible negatively charged mercaptosuccinic acid (MSA)-capped quantum dots (MSA-QDs) and positively charged polyethyleneimine-conjugated QDs (PEI-QDs) 80. The results showed that the negatively charged ultrasmall NPs accumulated gradually in the kidney, with only trace amounts of anionic QDs being detected in the urine. In contrast, PEI-QDs presented a more rapid renal clearance. This might be ascribed to the anionic GBM serving as a barrier, preventing the filtration of anionic QDs, rather than an increase in size resulting from protein binding.

### 2.3 Shape

The morphological characteristics of nanoplatforms exert profound influence on their biodistribution patterns through passive targeting mechanisms. Emerging evidence demonstrates that non-spherical architectures (e.g., rods, tubes, and sheets) exhibit enhanced cellular internalization compared to spherical counterparts, attributable to optimized membrane contact angles and increased surface-to-volume ratios 81. This geometric principle finds application in renal-targeted delivery systems, where flake-like DNA nanostructures demonstrate preferential kidney accumulation 82. Building upon this foundation, Hou *et al*. engineered black phosphorus nanosheets (BPNSs) - structurally analogous to DNA flakes - as reactive oxygen species (ROS) scavengers for treating AKI in murine models 83. These 2D nanomaterials effectively mitigated oxidative stress-induced apoptosis through renal-specific ROS elimination, highlighting the therapeutic potential of geometry-engineered nanocarriers.

### 2.4 Aspect ratio

Beyond geometric parameters, the aspect ratio of nanoplatforms governs their hemodynamic behavior and glomerular filtration dynamics. Higher aspect ratios not only promote margination but also facilitate interactions with endothelial cells lining blood vessels 84-86. It is worth noting that the unique physical properties of non-spherical nanoplatforms, particularly those with high aspect ratios, have significant implications for their behavior within the renal system. These nanomaterials possess the potential to evade traditional size-based clearance mechanisms due to their elongated shapes and may undergo filtration by the kidney in a manner distinct from spherical particles 87. Intriguingly, studies have shown that carbon nanotubes with lengths ranging from 200 to 300 nm can be effectively filtered by the GFB while maintaining their original configuration 88. This phenomenon is attributed to their orientation during passage through glomerular capillaries, where hydrostatic forces play a crucial role in aligning nanocarriers with high aspect ratios perpendicular to the GFB. As a result, these particles are able to insert their shorter axes into the slit diaphragm and subsequently undergo renal filtration. Moreover, non-spherical nanoplatforms pose a challenge for macrophages in terms of phagocytosis due to their irregular shapes and surface characteristics 89.

### 2.5 Density

The variations in buoyancy and circulation patterns arising from differences in densities can also notably impact their biodistribution, clearance rates, and targeting efficiency, notwithstanding the comparable size and charge. In actuality, the nanoplatforms of higher density tend to demonstrate greater buoyancy and do not remain at the center of the bloodstream where velocities are higher, but approach the vessel wall more rapidly. In contrast, low-density nanoplatforms circulate more rapidly in the bloodstream, giving rise to faster renal clearance, shorter blood retention times, and lower targeting 90, 91. For instance, substances of low density such as silica (2.32 g/cm^3^) exhibit sparing accumulation in the kidney and demonstrate significant renal excretion. Conversely, gold nanoplatforms with a material density of 19.32 g/cm^3^ are prone to display augmented buoyancy at endothelial openings, thereby leading to enhanced renal targeting and decreased renal clearance 92.

### 2.6 Surface modifications

The interaction between nanoplatforms and the biological barriers in the human body is a complex process that can significantly impact their performance. In addition to size, shape, charge, and density, the surface properties of nanomedicine play a crucial role in determining their fate within the body. The presence of various proteins in biological fluids can lead to the formation of a 'protein corona' on the surface of nanoplatforms. This corona effectively masks the original surface properties of the nanoplatform—such as surface charge, hydrophobicity, and functional ligands—and imparts a new biological identity that is recognized by cellular components and physiological systems 93. The inherent recognition capacity of the mononuclear phagocyte system presents a major biological barrier to nanoparticle-mediated drug delivery through rapid opsonization and subsequent clearance. To circumvent this limitation, surface engineering strategies employing polyethylene glycol (PEG) conjugation have been developed to prolong systemic circulation by sterically hindering protein adsorption and immune cell recognition 94. Demonstrating this principle, Heller and colleagues engineered poly(lactic-co-glycolic acid) (PLGA) nanoparticles through PEG functionalization, achieving sevenfold greater renal accumulation compared to non-modified counterparts 95. These nanoplatforms exhibited preferential localization in proximal tubular basal membranes rather than distal segments, with surface PEGylation simultaneously mediating controlled biodegradation kinetics and sustained therapeutic release. Mechanistic investigations revealed that peritubular capillary endothelial cells internalize nanoparticles through pressure gradient-driven endocytosis, facilitated by substantial transcapillary absorption forces within nephron microenvironments. This transport phenomenon underscores the critical interplay between nanocarrier surface engineering and physiological hydrodynamics in determining renal targeting efficiency.

### 2.7 Disease-related changes

Kidney diseases may arise from a diverse array of etiological factors, including genetic predispositions, autoimmune disorders, infectious agents, and exposure to nephrotoxic compounds. These pathological conditions frequently result in structural and functional impairments across multiple renal components, including the glomeruli, tubules, interstitium, and vasculature. Such impairments lead to significant alterations in the kidney's physiological milieu, thereby influencing the pharmacokinetics and biodistribution of therapeutically administered nanomedicines targeting renal tissues. Under normal physiological conditions, the GFB effectively restricts the passage of large macromolecules and anionic proteins into the urinary space. However, in pathological conditions such as AKI, CKD, and fibrotic nephropathy, structural alterations may compromise both the size and charge selectivity of the filtration barrier. Specifically, AKI-induced inflammation upregulates endothelial adhesion molecules while compromising glomerular filtration selectivity, thereby enhancing passive targeting *via* increased permeability 96, 97. In contrast, CKD exhibits progressive capillary rarefaction and collagen deposition that alter interstitial pressure gradients and macrophage-mediated nanoparticle clearance 98. Renal fibrosis presents unique extracellular matrix remodeling that generates size-selective transport barriers while overexpressing integrin receptors amenable to active targeting 99. These microenvironmental shifts collectively influence targeting efficiency, nanoparticles that are typically excluded due to their dimensions or surface charge may gain unintended access to the filtrate, while others may exhibit altered clearance patterns or aberrant accumulation as a result of modified permeability characteristics 100, 101.

## 3. Active renal targeted delivery strategy

**Figure 2:**
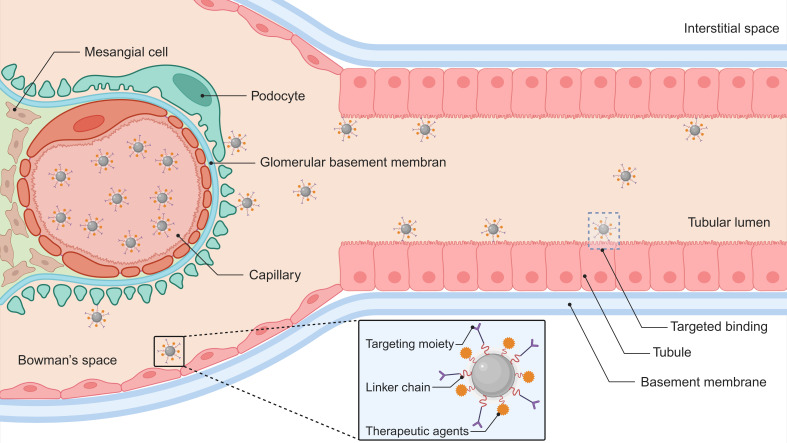
** Receptor-targeted therapeutic delivery nanoplatform.** This nanoscale architecture integrates three functional elements: (1) molecular recognition ligands for cellular specificity, (2) biodegradable chemical linkers, and (3) therapeutic agents. This system achieves spatial precision through ligand-receptor interactions with renal parenchymal membrane proteins, triggering energy-dependent endocytotic internalization. Created with Biorender.com.

Surface functionalization of nanomaterials with molecular recognition elements (e.g., antibodies, peptides, or small-molecule ligands) enhances renal specificity through ligand-receptor binding mechanisms (**Figure 2**). This targeting strategy promotes selective accumulation in critical nephron segments—particularly proximal tubules and glomerular cells. Compared to passive delivery systems, actively targeted nanocarriers demonstrate superior cellular internalization efficiency while minimizing extrarenal biodistribution 68, 102. Current research focuses on engineering these nanoplatforms for precise treatment of renal fibrosis, acute injury, and chronic inflammatory conditions, with experimental models showing substantially improved therapeutic effects (**Table 1**). Each targeting molecule presents distinct advantages and limitations, primarily dictated by the physicochemical properties of its key ligand and its cellular expression pattern (**Table S1**).

### 3.1 Kidney injury molecule-1 (KIM-1)

The pathophysiological microenvironment of AKI orchestrates dynamic molecular changes that drive both tissue repair and pathological progression. Among these regulatory factors, KIM-1, a transmembrane glycoprotein encoded by the HAVCR1 gene, has emerged as a critical mediator of renal epithelial remodeling. Originally identified by Ichimura *et al*. as T-cell immunoglobulin and mucin domain-containing protein 1 (TIM-1) 132, KIM-1 features a conserved structural architecture comprising an N-terminal immunoglobulin (Ig)-like domain with a hexacysteine motif and a mucin-rich extracellular region spanning residues 131-201. While KIM-1 expression is negligible in healthy renal tissue, its transcriptional activation becomes markedly upregulated in the proximal tubular S3 segment following ischemic or toxic insults. This spatial-temporal induction coincides with epithelial dedifferentiation, proliferation, and regeneration—a cascade essential for restoring functional nephron integrity. The ectodomain of KIM-1 contains O-linked and N-linked glycosylation sites within its threonine/serine/proline-enriched mucin domain, which facilitates ligand recognition and membrane stabilization 133, 134.

Intriguingly, the Ig-like domain confers specialized phagocytic capacity to injured tubular epithelial cells, enabling these cells to scavenge apoptotic debris *via* phosphatidylserine (PS) recognition. Mechanistically, the β-sandwich fold of the KIM-1 Ig domain binds PS, an aminophospholipid that translocates to the outer plasma membrane leaflet during apoptosis. This calcium-dependent interaction, mediated by a reverse-parallel β-strand topology analogous to C2 domains, positions KIM-1 as a semi-professional phagocytic receptor. Structurally, PS consists of a glycerol backbone esterified to two fatty acyl chains and a serine-linked phosphoryl headgroup. Its dynamic redistribution during apoptosis serves as an 'eat-me' signal, engaging KIM-1 to activate downstream pathways that mitigate inflammation while promoting epithelial repair. Structural modeling suggests that calcium coordination by the Ig domain enhances PS binding avidity, enabling selective recognition of damaged cells. These properties highlight KIM-1's dual role as a sentinel of tubular injury and a modulator of tissue homeostasis.

**Figure 3:**
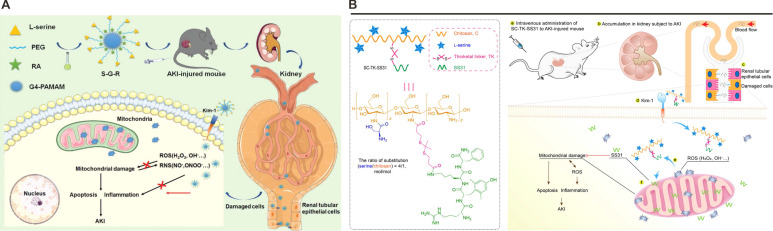
** Nanoplatforms targeting KIM-1.** (A) The structure of S-G-R and a schematic illustration of its mechanism for alleviating AKI injury. Adapted with permission from 107, copyright 2022 Wiley-VCH. (B) Chemical structure of SC-TK-SS31 and the schematic illustration showing the relief of AKI by SC-TK-SS31. Adapted with permission from 108, copyright 2020 American Association for the Advancement of Science.

The discovery that PS incorporates L-serine (Ser) residues—bearing hydroxyl (-OH), amino (-NH₂), and carboxyl (-COOH) functional groups—has inspired innovative strategies for kidney-specific drug delivery. Pioneering work by Yamamoto *et al*. in 2018 demonstrated this concept by synthesizing Ser-conjugated polyamidoamine (PAMAM) dendrimers (G3) for AKI-targeted therapy 105. Pharmacokinetic analyses revealed that Ser-PAMAM achieved 82% renal accumulation in murine models within 3 h post-injection, outperforming threonine- and tyrosine-modified analogs (34% and 9%, respectively). This specificity arises from the Ser side chain's optimal spatial arrangement: its single methylene spacer enables precise interaction with KIM-1's Ig domain, whereas bulkier residues impair binding. Near-infrared imaging and single-photon emission computed tomography (SPECT/CT) corroborated preferential renal localization, underscoring Ser's utility as a targeting ligand. Subsequent studies expanded this paradigm by integrating Ser into advanced nanocarrier systems. For instance, fourth-generation PAMAM dendrimers (G4) conjugated to Ser and loaded with rosmarinic acid (RA) generated S-G-R nanoparticles, which exploited glomerular endothelial pore permeability during AKI to bypass the intact GBM (**Figure 3**A) 107. Under ischemic conditions, disrupted GBM charge selectivity permitted S-G-R extravasation into proximal tubules, where KIM-1-mediated endocytosis enhanced RA delivery to injured epithelia.

Parallel innovations include the development of Ser-modified chitosan (SC) combined with the mitochondrial-targeting peptide SS31 (**Figure 3**B) 108. Leveraging chitosan's cationic charge for renal retention, the SC-TK-SS31 conjugate incorporated a thioketal (TK) linker for ROS-responsive drug release. *In vitro* and *in vivo* studies confirmed mitochondrial accumulation and prolonged therapeutic action in AKI models. Collectively, these findings establish KIM-1 as a potential target for next-generation renal therapeutics. By leveraging its PS-binding specificity, Ser-modified nanoplatforms achieve excellent renal targeting efficiency in preclinical models, but their clinical translation necessitates rigorous optimization of pharmacokinetic parameters, including biodistribution profiles and clearance mechanisms. Future investigations should prioritize mechanistic studies to delineate KIM-1's cross-talk with intrinsic repair pathways, such as Hippo/YAP signaling or metabolic reprogramming events, which may synergize with nanoparticle-mediated therapies. While these developments highlight KIM-1's expanding role beyond conventional biomarker applications, its full potential as a pivotal molecular target for precision medicine in kidney disease remains robust validation across diverse etiologies of kidney injury.

### 3.2 Melanocortin receptor 1 (MC1R)

MC1R, a G protein-coupled receptor, has emerged as a critical molecular target for renal therapy due to its unique expression profile and signaling properties in kidney cells. Berg and colleagues first implicated MC1R in kidney physiology through the serendipitous observation that ACTH, a non-selective melanocortin agonist, reduced proteinuria in patients with membranous nephropathy 135. Subsequent expression profiling confirmed MC1R as the predominant melanocortin receptor in the human renal cortex, with the highest density localized to podocytes. This renal-specific expression pattern underlies the therapeutic rationale for MC1R-targeted interventions.

Mechanistically, MC1R activation in podocytes initiates a protective cascade mediated by cAMP-PKA signaling. Under pathological conditions such as puromycin-induced injury, MC1R upregulation correlates with increased intracellular cAMP levels, which in turn enhance catalase activity, reduce ROS, and promote p190RhoGAP dephosphorylation. These molecular events stabilize podocyte cytoskeletal architecture through RhoA modulation, counteracting stress fiber disruption and apoptosis 136, 137. Experimental validation in Heymann nephritis models demonstrated that selective MC1R agonism with MS05 significantly attenuates proteinuria and preserves glomerular ultrastructure, confirming the receptor's functional significance in renal pathophysiology 138.

**Figure 4:**
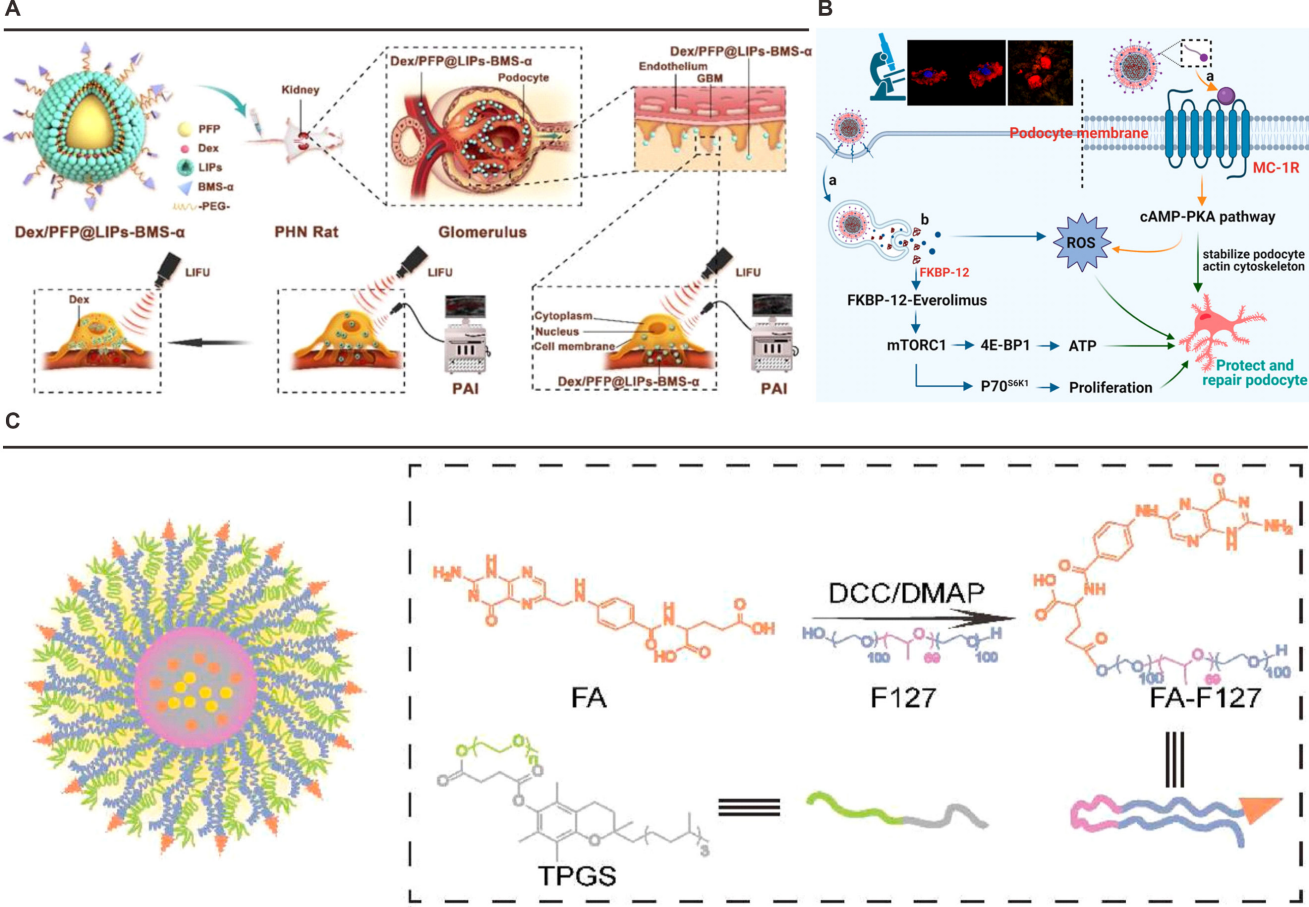
** Nanoplatforms targeting MC1R or FR-α.** (A) Schematic illustration of the structure and theranostic functions of Dex/PFP@LIPs-BMS-α. Adapted with permission from 111, copyright 2021 Ivyspring International Publisher. (B) The specific podocyte-targeting mechanism of ESC-HCM-B and a schematic illustration of its action in alleviating podocyte injury under high-glucose conditions. Adapted with permission from 112, copyright 2023 American Chemical Society. (C) Schematic illustration of Cur/Res@FA-F127/TPGS NPs and their functional mechanism. Adapted with permission from 113, copyright 2022 Elsevier.

The development of MC1R-targeted nanotherapeutics capitalizes on this receptor-ligand specificity to achieve renal precision. BMS-470539 (BMS-α), a synthetic MC1R agonist with high binding affinity. For instance, Dex/PFP@LIPs-BMS-α exploits this targeting mechanism through surface-conjugated BMS-α, enabling selective recognition of podocyte MC1R (**Figure 4**A) 111. This strategy overcomes the glomerular filtration barrier's physiological constraints, as demonstrated by transmission electron microscopy showing predominant nanoparticle accumulation in podocyte layers. The system's ultrasound-responsive perfluoropentane core further enhances spatial control through triggered drug release at target sites.

Complementary approaches integrate MC1R's anti-inflammatory properties with antioxidative therapies. The ESC-HCM-B nanoparticle platform conjugates BMS-α to mesoporous silica cores loaded with everolimus and cerium dioxide quantum dots (**Figure 4**B) 112. This dual-action system simultaneously addresses mTOR pathway dysregulation and oxidative stress, while leveraging MC1R-mediated cAMP signaling to amplify cytoprotective effects. Importantly, biodistribution studies confirm renal specificity, with minimal hepatic or splenic sequestration, underscoring the efficiency of MC1R-driven targeting.

The translational potential of MC1R-based strategies is further supported by its pharmacodynamic advantages. Despite lower receptor density in renal cells compared to melanocytes, MC1R exhibits picomolar sensitivity to agonists in podocytes, enabling therapeutic effects at minimal dosages. This enhanced signaling efficiency, coupled with the receptor's intrinsic role in countering inflammation and oxidative damage, provides a multifaceted therapeutic framework for proteinuric kidney diseases. Current challenges center on optimizing ligand-receptor binding kinetics and improving nanoparticle penetration through sclerotic glomeruli, areas requiring further structural refinement of both targeting moieties and delivery vehicles.

### 3.3 Folate receptor α (FR-α)

The therapeutic exploitation of FR-α for renal targeting hinges on its unique biochemical duality: unparalleled renal overexpression and evolutionarily conserved ligand-binding architecture. Quantitative tissue profiling demonstrates FR-α density in proximal tubules exceeds hepatic levels by 80-fold, a disparity magnified by the receptor's picomolar folate affinity that compensates for lower renal folate concentrations. Physiological studies using isoform-specific knockouts established FR-α's irreplaceable role in tubular reabsorption, with FR-β deficiency causing negligible functional impairment 139-144. This functional hierarchy, coupled with FR-α's polarized apical localization in tubular epithelium, creates an exploitable interface for renal-targeted drug delivery.

Structural elucidation of FR-α reveals design principles enabling its targeting efficacy. Crystallographic analyses identify a conserved ligand-binding cleft (10 × 15 × 20 Å) stabilized by eight disulfide bonds (C15-C43, C35-C83, C44-C87, C67-C153, C74-C124, C113-C187, C117-C167, C130-C147) critical for maintaining tertiary structure during endocytic recycling 145, 146. The pterin moiety anchors through π-stacking with Y85/W171 (3.3-3.7 Å) and hydrogen bonding to Y175 (2.8 Å), burying 89% of its surface area within a hydrophobic pocket. Simultaneously, the aminobenzoate group engages W102/W134 *via* CH-π interactions (3.4 Å), while glutamate carboxyls form six hydrogen bonds (2.8-3.2 Å) with K136, W140, and H135-G137-W138 clusters. Remarkably, β1-β2 loop flexibility permits 12º conformational shifts accommodating macromolecular conjugates without significant affinity loss.

These structural insights directly inform nanotechnology development. Leamon's foundational work established γ-glutamyl carboxylates as optimal conjugation sites, with steric tolerance extending 14.3 Å beyond the p-aminobenzoate core—parameters enabling stable nanoparticle linkage while preserving FR-α engagement. Huang *et al*.'s folate-conjugated Pluronic nanoparticles (FPNPs) operationalize this principle, demonstrating 7.2-fold greater renal AUC_0-48h_ versus non-targeted counterparts through FR-mediated endocytosis 147. Real-time tracking reveals biphasic accumulation: rapid glomerular extravasation (T_max_ = 12 min) followed by sustained tubular uptake peaking at 12 h. In ischemia-reperfusion models, FPNP-delivered tretinoin lactone alcohol reduces acute tubular necrosis scores from 3.8 ± 0.4 to 1.3 ± 0.2 while lowering serum creatinine by 78%—effects unattainable with free drug administration.

The strategy's adaptability extends to multi-mechanistic systems addressing complex pathophysiology. Du *et al*.'s hybrid micelles (Cur/Res@FA-F127/TPGS) exploit FR-α's rapid internalization cycle to deliver synergistic anti-inflammatory payloads, combining resveratrol's SIRT1 activation with curcumin's NF-κB inhibition (**Figure 4**C) 113. Computational simulations confirm the system's spatial confinement to renal tissue correlates with FR-α's ligand-binding plasticity, accommodating varied therapeutic architectures through conserved tryptophan clusters (W102, W134, W140).

The evolving understanding of renal folate transport mechanics—particularly FR-α's dual role in tubular reabsorption and therapeutic targeting—provides a biochemical blueprint for precision nephrology. While current nanoplatforms demonstrate functional efficacy in ischemia models, persistent challenges in endosomal escape efficiency demand molecular-scale engineering solutions. Future innovation should focus on β1-β2 loop-adaptive nano-constructs and machine learning-guided ligand optimization to surmount lysosomal entrapment. Validating these advances in humanized models will bridge the translational gap, ultimately enabling multi-mechanistic therapies for progressive glomerulopathies.

### 3.4 Megalin receptor

Megalin, a membrane-associated endocytic receptor predominantly expressed in renal proximal tubular epithelial cells, plays a central role in receptor-mediated reabsorption of glomerular filtrate components 148, 149. This high-molecular-weight (600 kDa) transmembrane glycoprotein of the low-density lipoprotein (LDL) receptor family, initially characterized as the pathogenic antigen in Heymann nephritis, demonstrates critical structural specialization for ligand recognition and intracellular trafficking. The receptor's extracellular architecture contains four ligand-binding clusters comprising 36 cysteine-enriched complement-type repeats, 16 growth factor repeats with pH-sensitive YWTD spacers, and an epidermal growth factor (EGF)-like domain. Notably, the 209-amino-acid cytoplasmic tail contains two evolutionarily conserved NPXY endocytic motifs that orchestrate vesicular transport through interactions with clathrin adaptor AP-2 and bridging proteins (Dab2, ARH). A distinctive NQNY motif further directs apical membrane localization, while uncharacterized SH3/PDZ domains suggest additional regulatory potential.

The receptor's strategic positioning at the brush border membrane of proximal tubular epithelial cells enables its pivotal function in reclaiming filtered proteins, evidenced by seminal studies using 125I-labeled albumin 150. Ligand-competition experiments with receptor-associated protein (RAP) demonstrated 80% inhibition of albumin uptake, establishing megalin as the principal mediator of tubular protein recovery. This endocytic specialization has been innovatively leveraged in nanotechnology: the BSA@PDA@Fe nanoprobe exemplifies precise megalin-mediated renal targeting through structural mimicry of endogenous ligands 114. The 2.7 nm construct combines BSA-mediated receptor recognition with PDA-derived antioxidant protection, demonstrating pH-responsive ROS scavenging and reduced cellular oxidative damage in HK-2 models.

**Figure 5:**
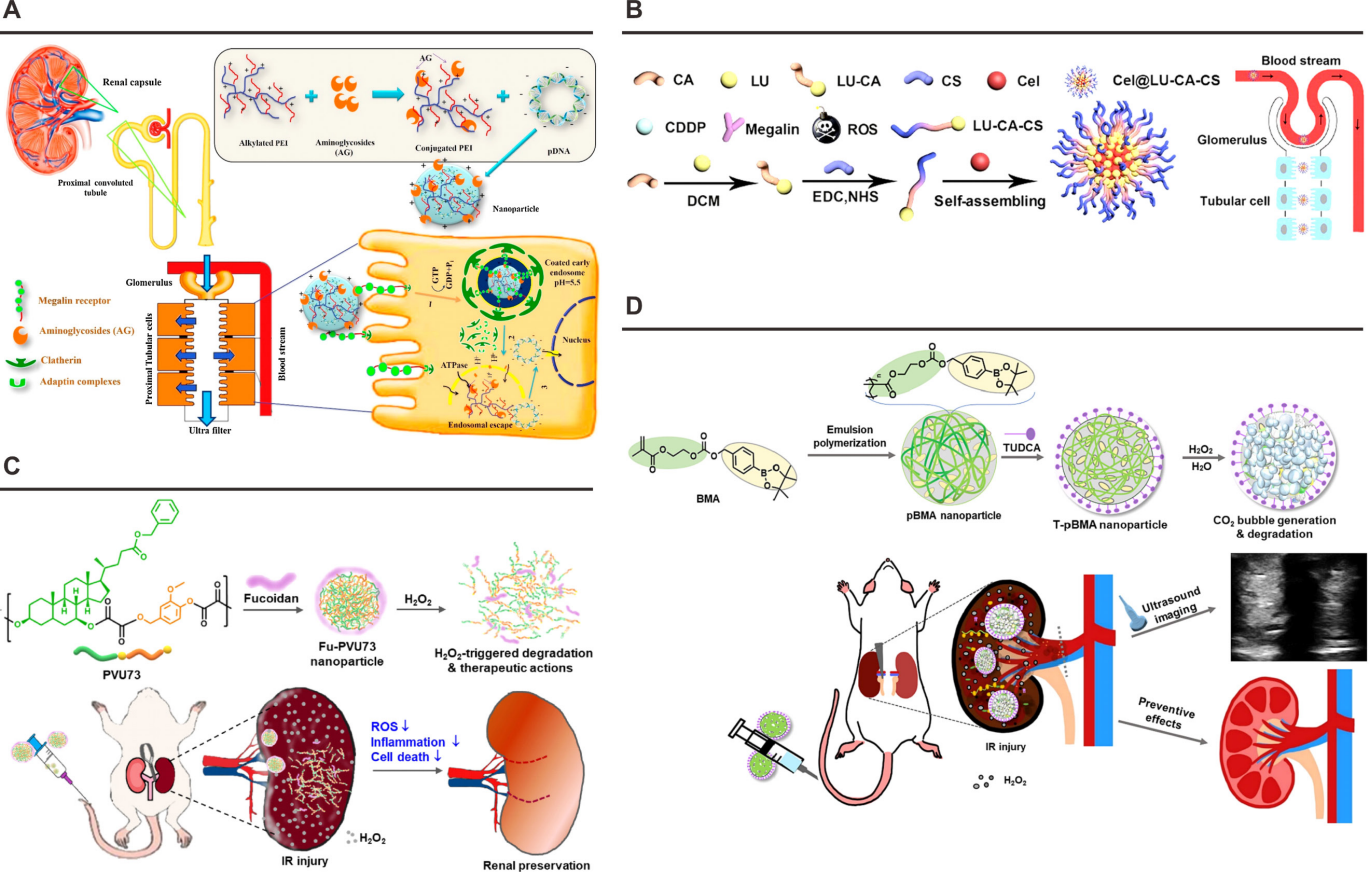
** Nanoplatforms targeting the Megalin Receptor or P-selectin.** (A) Schematic illustration of the structure and cytoprotective functions of polymyxin-PEI/DNA. Adapted with permission from 116, copyright 2017 Elsevier. (B) Synthesis and renal protective mechanism of the Cel@LU-CA-CS nanoplatform. Adapted with permission from 117, copyright 2022 Elsevier. (C) Schematic illustration of the Fu-PVU73 nanoplatform and its functional mechanism for renal preservation. Adapted with permission from 119, copyright 2022 American Chemical Society. (D) Schematic illustration of T-pBMA nanoparticles reacting with overproduced H₂O₂ to enhance ultrasound contrast, while exerting potent antioxidant, anti-inflammatory, and antiapoptotic effects. Adapted with permission from 121, copyright 2023 Elsevier.

Mechanistic investigations of nephrotoxin uptake further validate megalin's therapeutic relevance. Aminoglycoside accumulation studies reveal concentration-dependent receptor saturation kinetics in S1/S2 tubular segments, providing molecular rationale for drug-induced nephropathy. Targeted intervention strategies exploit this pathway through ligand-receptor engineering: covalent conjugation of gentamicin/neomycin to carboxyalkylated PEI polymers created polyplexes demonstrating megalin-specific transfection enhancement (**Figure 5**A) 116. PEI25 derivatives achieved 13-fold increased EGFP expression in HK-2 cells versus controls, with strict megalin-dependence confirmed through comparative studies in receptor-deficient HepG2 lines. Parallel development of polymyxin B-PEI conjugates yielded analogous targeting efficacy while reducing PEI-associated cytotoxicity, confirming structural modularity of this delivery paradigm 115. Structural analysis of chitosan derivatives revealed intrinsic megalin affinity attributable to conserved aminoglycoside motifs. Pharmacokinetic profiling of N-acetylated low-molecular-weight chitosan (LMWC) conjugates demonstrated 13-fold renal accumulation versus free drug controls, with megalin-knockout models confirming receptor-mediated uptake 151. Advanced nanoplatforms incorporating pH-sensitive chitosan derivatives (Cel@LU-CA-CS) synergize megalin targeting with microenvironment-responsive drug release, addressing oxidative stress through spatial control of antioxidant delivery (**Figure 5**B) 117. Such multifunctional systems exemplify the translational potential of receptor-based engineering in precision nephrology.

This body of work establishes megalin as a master regulator of renal transport physiology and a promising target for molecular therapeutics. Continued refinement of ligand-receptor interaction dynamics, coupled with innovations in nanostructure design, positions megalin-mediated delivery as a cornerstone strategy for overcoming biological barriers in renal disease management.

### 3.5 P-selectin

Renal ischemia-reperfusion (I/R) injury represents a principal contributor to acute renal failure pathogenesis, with dysregulated neutrophil recruitment emerging as a critical patho-mechanism 152. The selectin family of calcium-dependent vascular adhesion molecules, particularly P-selectin, has been mechanistically implicated in this process through its regulation of leukocyte-endothelial interactions. Structurally characterized by conserved N-terminal C-type lectin domains flanked by epidermal growth factor-like motifs and short consensus repeats (SCRs), selectins coordinate multistep leukocyte trafficking *via* specialized domain-exon correspondence—a genomic architectture preserving functional modularity across species.

P-selectin's pathophysiological dominance in renal I/R injury was first evidenced through blockade experiments demonstrating attenuated neutrophil infiltration and preserved renal function. This transmembrane glycoprotein exhibits rapid surface mobilization from endothelial Weibel-Palade bodies and platelet α-granules upon inflammatory activation, initiating leukocyte rolling through calcium-dependent interactions with PSGL-1. The mucin-type ligand PSGL-1 requires precise post-translational modifications for functional competence: core-2 β-1,6-N-acetylglucosaminyltransferase-mediated carbohydrate branching, tyrosine sulfation at three critical NH2-terminal residues, and sialyl Lewis x epitope presentation 153. This molecular understanding has catalyzed innovative targeting strategies. The FU-PVU73 nanotherapeutic exemplifies precision engineering through dual-pathology intervention: fucoidan surface decoration confers P-selectin affinity *via* competitive PSGL-1 mimicry, while persulfate crosslinkers enable H₂O₂-responsive payload release of ursodeoxycholic acid and vanillin derivatives (**Figure 5**C) 119. Pharmacodynamic evaluations demonstrate concurrent blockade of neutrophil adhesion (IC_50_ = 2.3 nM) and ROS scavenging capacity (12-fold *vs* free drugs) in ischemia-challenged endothelia. In a separate investigation, arylboronate-containing poly(methacrylate) (pBMA) was synthesized as a H_2_O_2_-triggerable polymeric prodrug with dual antioxidant and antiapoptotic capacities (**Figure 5**D) 121. Subsequently, the surface of pBMA nanoparticles was functionalized with taurodeoxycholic acid (TUDCA), which specifically targets P-selectin that is overexpressed in inflammatory lesions. In a murine model of renal I/R injury, TUDCA-coated pBMA (T-pBMA) nanoparticles demonstrated preferential accumulation in the injured kidney and mitigated renal I/R injury through efficient scavenging of H_2_O_2_ and downregulation of inflammatory cytokine expression.

Emerging structure-activity relationships highlight the critical role of fucose spatial presentation. Molecular dynamics simulations suggest optimal P-selectin engagement requires multivalent clustering of α1-3 linked fucose residues within 4.2 Å spacing, a configuration faithfully replicated in fucoidan-based carriers 154, 155. This geometric precision ensures superior renal accumulation in LPS-induced AKI models, as evidenced by quantitative biodistribution studies showing a significantly higher kidney uptake for optimized micelles compared to non-targeted counterparts. Current limitations persist regarding ligand-receptor saturation kinetics and systemic complement activation risks. Recent progress in click chemistry-mediated ligand optimization and glycocalyx-targeting strategies demonstrates potential to address these challenges, as the integration of computational ligand design with responsive nanoplatform engineering enables enhanced control of renal inflammation.

### 3.6 CD44

CD44, a multifunctional transmembrane glycoprotein integral to angiogenesis, immune regulation, and cellular dynamics, operates as a principal molecular orchestrator of cell-matrix communication 156. Its functional architecture comprises four distinct structural elements: an N-terminal signal peptide, an extracellular ligand-binding domain containing conserved glycosaminoglycan recognition motifs, a transmembrane α-helix, and an intracellular C-terminal segment that mediates cytoskeletal coupling through ezrin-radixin-moesin protein interactions 157. The extracellular domain employs a sophisticated recognition system, where three cysteine-rich subdomains (Cys81-Cys101) form a specialized hyaluronic acid (HA) binding pocket, while post-translational modifications including PKC-mediated phosphorylation and S-palmitoylation dynamically regulate cytoplasmic domain signaling. At the molecular level, CD44 manifests its pathophysiological significance through high-affinity interactions with HA, a megadalton glycosaminoglycan polymer composed of alternating D-glucuronic acid and N-acetyl-D-glucosamine units. Crystallographic analyses reveal a dual-mode binding mechanism: hydrophobic interactions between Tyr83-Ile92 residues stabilize HA backbone accommodation, while a network of hydrogen bonds between HA carboxyl groups and Arg45/Tyr46 side chains ensures subnanomolar affinity (K_d_ = 0.3 nM). This molecular recognition event initiates signal transduction cascades that coordinate Nanog-mediated multidrug resistance regulation, JNK pathway activation, and GSK3β-mediated β-catenin degradation 158-161.

**Figure 6:**
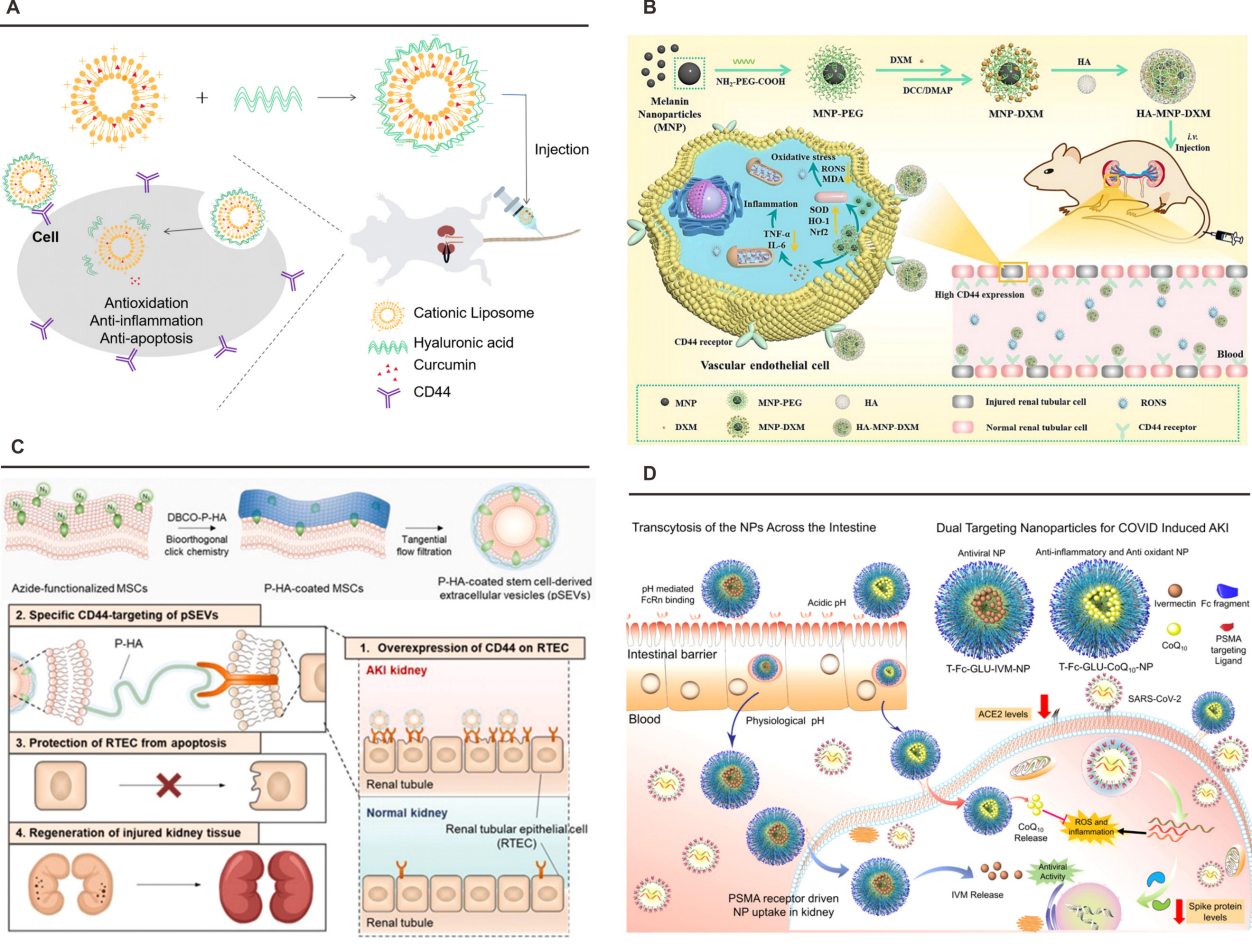
** Nanoplatforms targeting CD44 or PSMA.** (A) Schematic illustration of pH-sensitive HA-liposome hybrids enhancing curcumin bioavailability in acidic AKI milieus. Adapted with permission from 122, copyright 2023 Elsevier. (B) The preparation route and schematic illustration of targeting HA-MNP-DXM NPs for treating I/R-induced AKI through scavenging RONS and alleviating inflammation-induced damage. Adapted with permission from 124, copyright 2024 Elsevier. (C) The approach for editing the surface of SEVs *via* metabolic glycoengineering-mediated click chemistry, and the effect of pSEVs on RTECs in an AKI model. Adapted with permission from 162, copyright 2025 Elsevier. (D) FcRn-mediated transcytosis across the gut epithelial barrier, subsequent circulation and localization in the kidney *via* PSMA-mediated uptake into kidney cells, and release of IVM and CoQ10 to reduce ACE2 and spike protein expression, as well as mitigate inflammation and ROS levels in kidney cells. Adapted with permission from 125, copyright 2021 American Chemical Society.

Therapeutic exploitation of CD44-mediated targeting capitalizes on its pathophysiological overexpression during ischemic AKI, were peritubular HA deposition signals tissue regeneration. Nanoparticle surface engineering with HA oligosaccharides achieves 12.8-fold enhancement in renal accumulation through receptor-mediated endocytosis, as validated by competitive inhibition assays demonstrating 73% reduction in nanoparticle retention following HA pretreatment. Advanced delivery systems incorporate microenvironment-responsive elements, exemplified by pH-sensitive HA-liposome hybrids that enhance curcumin bioavailability 4.2-fold in acidic AKI milieus, correlating with 58% reduction in serum creatinine compared to free drug administration (**Figure 6**A) 122. Designs integrate therapeutic payloads with ROS-scavenging functionality, as demonstrated by HA-conjugated melanin nanoparticles that simultaneously deliver dexamethasone and neutralize 92% of reactive oxygen species in ischemia-reperfusion models, restoring Nrf2/HO-1 axis functionality to achieve 86% survival versus untreated controls (**Figure 6**B) 124. In addition, Kim *et al*. developed engineered extracellular vesicles (pSEVs) derived from PEGylated hyaluronic acid-modified mesenchymal stem cells using metabolic glycoengineering-mediated click chemistry. In cisplatin-induced AKI models, pSEVs achieved targeted kidney accumulation through HA-CD44 and HA-Toll-like receptor 4 interactions, significantly attenuating renal dysfunction markers and retarding organ weight loss (**Figure 6**C) 162.

Clinical translation studies validate the therapeutic index of CD44-targeted systems through human proximal tubule models demonstrating 94% renal specificity. A critical advancement involves glutathione-responsive payload release systems that achieve excellent control, delivering 83% therapeutic cargo within 8 h under pathological pH conditions while minimizing off-target effects. Emerging platforms leverage HA-modified exosomes for CRISPR-Cas9 delivery in phase II clinical trials, targeting monogenic nephropathies through precision genome editing. This molecular targeting paradigm exemplifies the convergence of structural biology and nanomedicine, establishing CD44 as a keystone receptor in renal therapeutics while demonstrating transformative potential in precision nephrology through progressive refinement from basic ligand-receptor characterization to clinical-grade delivery systems.

### 3.7 Prostate-specific membrane antigen (PSMA)

PSMA, a type II transmembrane glycoprotein, has emerged as a pivotal renal targeting receptor due to its distinctive spatial expression and catalytic architecture. Baccala *et al.* identified its exclusive localization to proximal tubule brush borders in normal kidneys, with absent expression in glomerular or vascular components 163. The conserved predominance of its glutamate carboxypeptidase II activity in these tubules across species underlines its role as a renal-selective molecular address. Structurally, PSMA features a truncated 19-residue intracellular domain, 24-amino acid transmembrane anchor, and an expansive 707-amino acid extracellular domain harboring enzymatic activity 164. Its dual functionality as folate hydrolase and neuropeptidase drives therapeutic targeting strategies, first enabled through 7E11 monoclonal antibody detection in prostate cancer models 165.

The molecular recognition mechanism of PSMA revolves around zinc-mediated coordination chemistry 166. Urea-based inhibitors like Lys(β-naphthylalanine)-NH-CO-NH-Glu (Lys(Nal)Urea-Glu) exploit this configuration: three carboxyl groups from the Glu-Urea-Lys motif form charge-complementary interactions with active site residues, while the urea oxygen chelates the catalytic zinc ion. Hydrophobic stabilization *via* naphthylalanine aromatic rings completes this precision binding, establishing glutamate-urea-R (Glu-Urea-R) derivatives as optimal targeting ligands 164, 167. These engineered molecules demonstrate enhanced metabolic stability, tissue penetration, and renal tropism, enabling their integration into nanoparticle surfaces through glutaric acid urea analogs. The resultant conformation-specific targeting systems achieve spatial control over drug delivery, particularly valuable in overcoming biological barriers.

This targeting paradigm has been effectively operationalized in Bapurao Surnar *et al*.'s dual-functional nanoplatform (**Figure 6**D) 125. The system synergizes two PLGA-PEG copolymers: PLGA-*b*-PEG2000-Mal targets intestinal FcRn receptors to enhance systemic bioavailability, while PLGA-*b*-PEG3500-GLU engages renal PSMA *via* glutamate-urea interactions. Co-encapsulation of ivermectin and coenzyme Q10 facilitates concurrent antiviral activity against SARS-CoV-2 and mitigation of kidney injury markers. Preclinical validation confirmed successful negotiation of intestinal and renal epithelial barriers, with PSMA-mediated endocytosis driving preferential renal accumulation. The optimized formulation reduced ACE2 expression by 62%, suppressed viral spike protein synthesis, and attenuated oxidative stress through ROS reduction, underscoring the clinical viability of PSMA-targeted delivery.

The strategic exploitation of PSMA's biological properties through nanoscale engineering marks a paradigm shifts in renal therapeutics. By capitalizing on its restricted tubular expression and catalytic architecture, surface-modified nanoparticles achieve unparalleled renal specificity while evading off-target effects. Current investigations focus on refining ligand-receptor interaction kinetics through stereochemical optimization and multiplexed targeting modalities. Such advancements promise to transform therapeutic approaches for chronic kidney disease, drug-induced nephrotoxicity, and viral-associated renal pathologies, ultimately enabling precision interventions tailored to individual molecular profiles. This convergence of structural biology and nanomedicine positions PSMA-targeted systems at the forefront of next-generation renal therapeutics.

PSMA-directed nanoengineering exhibits promise in renal therapeutics through tubular localization-guided targeting and catalytic functionality, with surface-modified nanoparticles demonstrating enhanced tissue selectivity. Future research may further optimize these platforms by investigating ligand-receptor interaction kinetics and combinatorial targeting paradigms, potentially enabling precision nanomedicine solutions for renal disorders through interdisciplinary biological-engineering synergies.

### 3.8 Vascular cell adhesion molecule-1 (VCAM-1)

Glomerular endothelial cells serve as primary vascular gatekeepers, maintaining selective permeability through surface glycocalyx and charge barriers. Pathological disruption of these cells initiates proteinuria and inflammatory cascades, during which VCAM-1 undergoes marked upregulation. This inflammation-induced VCAM-1 overexpression facilitates receptor-mediated nanocarrier internalization, positioning it as a strategic target for renal inflammatory therapeutics 168. Effective exploitation of VCAM-1's therapeutic potential necessitates detailed understanding of its structural architecture and ligand-binding mechanics.

Characterized as a transmembrane immunoglobulin superfamily member, VCAM-1 comprises seven extracellular Ig-like domains with tandem repeat symmetry (domains 1-3 mirroring 4-6) 169. Functional analyses reveal domain 1/4 as primary binding interfaces, while adjacent domains 2/5 maintain structural integrity. Its principal ligands, α4β1 (VLA-4) and α4β7 integrins expressed on leukocyte subsets, mediate physiological adhesion events preceding transmigration. This interaction operates bidirectionally: inactive α4β1 mediates endothelial rolling, while chemokine-activated conformations enable high-affinity binding *via* VCAM-1's N-terminal cysteine residues. Crystallographic studies of VCAM-1's N-terminal D1D2 fragment reveal two immunoglobulin folds—domain 1 exhibiting a group I fold with unique C-D loop architecture stabilized by Asp-40 hydrogen bonding, and domain 2 adopting an extended C2 fold with flexible loops. Notably, an auxiliary Cys28-Cys75 disulfide bond in domain 1 induces apex compaction, creating topological complementarity for integrin binding 170.

**Figure 7:**
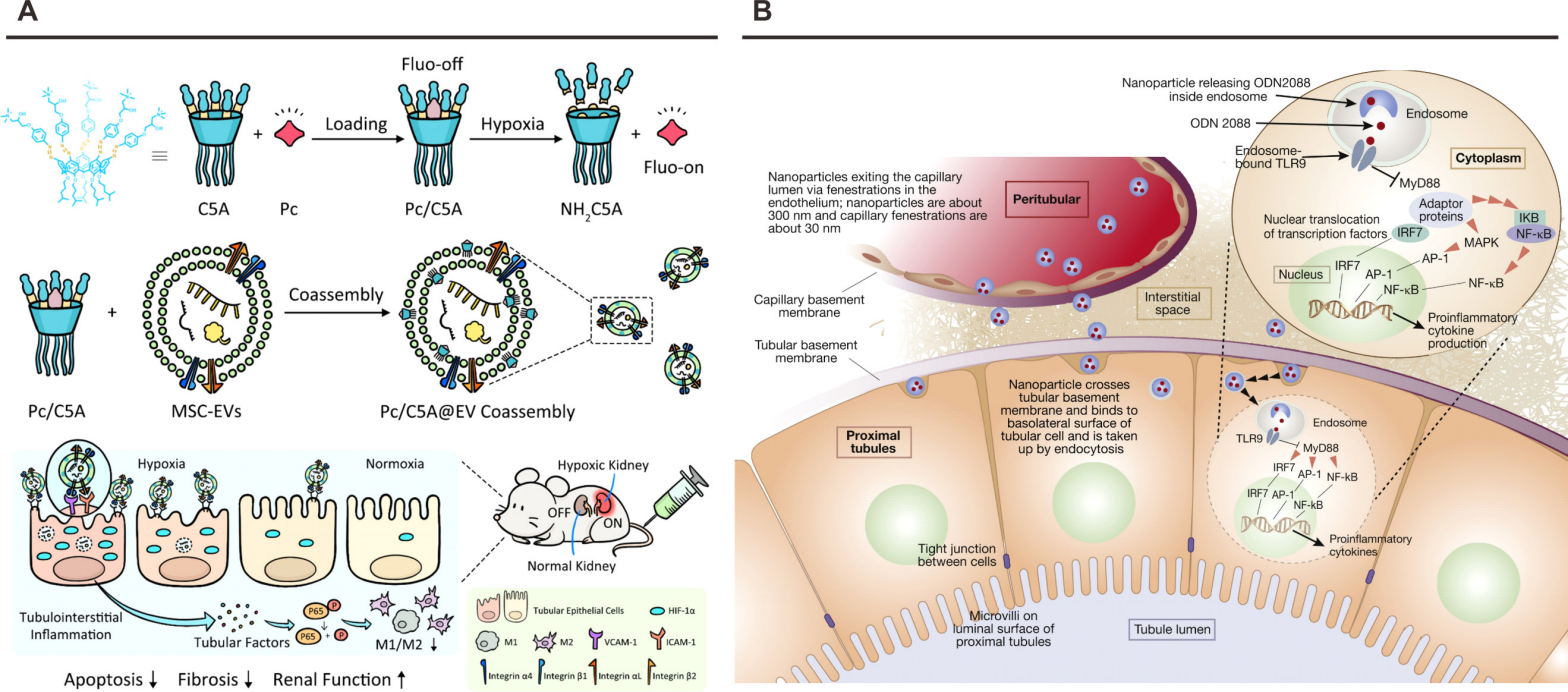
** Nanoplatforms targeting VCAM-1 or TLR9.** (A) Schematic illustration of Pc/C5A@EV preparation and its application for simultaneous hypoxia-sensitive imaging and therapy in injured kidneys. Adapted with permission from 126, copyright 2021 Springer Nature. (B) Schematic illustration of nanoparticles delivering ODN2088 to proximal tubular cells. Adapted with permission from 180, copyright 2020 Elsevier.

The D1-D2 interface demonstrates interdomain flexibility mediated by Tyr-89, permitting 12° rotational variation between molecules. This plasticity contrasts with homologous CD4-D1D2 systems, where β-barrel orientations remain parallel. Functional mapping localizes critical binding activity to domain 1's Asp-40 within the C-D loop—a structural motif recapitulated in cyclic peptide mimetics showing superior α4β1 inhibition compared to linear counterparts. These insights guide rational engineering of VCAM-1-targeted nanoplatforms exploiting integrin binding kinetics. Modern therapeutic strategies leverage VCAM-1's inflammation-specific expression through multiple modalities. Mesenchymal stem cell-derived extracellular vesicles (MSC-EVs) demonstrate natural tropism *via* surface α4β1/αLβ2 integrins engaging VCAM-1 on ischemic endothelia. Cheng *et al*. augmented this targeting through hypoxia-responsive Pc/C5A@EV complexes, where host-guest chemistry extends circulation while integrin-VCAM-1 interactions direct renal accumulation (**Figure 7**A) 126. Parallel innovations include peptide-conjugated platforms like Wu's PC-PLNS—110 nm phospholipid nanoparticles (+12 mV ζ-potential) functionalized with VHPKQHR motifs 171. These systems exploit VCAM-1's conformational epitopes through GGSKGC-linked peptides, achieving dual active-passive targeting of inflamed glomeruli. Celastrol-loaded PC-PLNS exhibit preferential uptake by damaged endothelia/podocytes, demonstrating enhanced therapeutic precision in chronic kidney disease models.

The structural elucidation of VCAM-1's dynamic Ig domains and integrative binding mechanisms provides a blueprint for next-generation renal nanotherapeutics. Future developments may incorporate machine learning-driven peptide optimization and multiplexed targeting strategies to address pathological heterogeneity. As these systems progress, rigorous assessment of endothelial activation thresholds and long-term biocompatibility will be essential to translate structural insights into clinically viable precision therapies.

### 3.9 Toll-like receptor 9 (TLR-9)

TLR-9, as a member of the type I transmembrane protein family, is characterized by three critical structural domains: an extracellular leucine-rich repeat (LRR) ligand-binding domain, a transmembrane domain, and an intracellular Toll/IL-1 receptor (TIR) signaling domain 172. The extracellular LRR domain forms a solenoid structure through 24-residue tandem repeats, featuring parallel β-sheets on the concave surface and α-helical elements interspersed with unstructured loops on the convex surface. Of the 10 human TLR subtypes identified, TLR-9 has emerged as a pivotal mediator in renal pathophysiology. Initial evidence from Benigni *et al*. established TLR-9 activation in tubular epithelial cells as a pathogenic driver of tubulointerstitial inflammation in murine and human lupus nephritis models 173. This finding was subsequently corroborated by Papadimitraki *et al.*, who demonstrated TLR-9 overexpression in glomerular compartments of lupus nephritis patients, with endogenous nucleic acid ligands triggering proinflammatory signaling in glomerular cells 174. Building upon these observations, Han *et al.* delineated the dualistic role of renal TLR-9 in I/R induced AKI, revealing that tubular TLR-9 activation exacerbates parenchymal damage through NF-κB-mediated inflammation and caspase-dependent apoptosis, whereas extra-tubular TLR-9 signaling paradoxically confers cytoprotection. This cellular compartment-specific functionality underscores the therapeutic rationale for spatial precision in TLR-9 modulation 175.

The molecular basis of TLR-9 activation lies in its capacity to distinguish bacterial DNA from host genetic material. While mammalian DNA maintains extensive CpG dinucleotide methylation, bacterial genomes exhibit hypomethylated CpG motifs that serve as pathogen-associated molecular patterns 176. Synthetic oligonucleotides containing unmethylated CpG sequences (CpG-ODNs) recapitulate these immunostimulatory effects, with optimal murine B-cell activation achieved through sequences conforming to the 'non-C, unmethylated C, G, non-G' paradigm 177-179. Mechanistically, TLR-9 directly engages CpG-ODNs through its extracellular domain, with binding specificity dictated by sequence composition. This interaction is competitively inhibited by G-quadruplex-forming ODNs such as ODN2088 and ODN2296, which suppress CpG signaling by occupying the TLR-9 ligand-binding pocket without interfering with cellular uptake. Structural analyses reveal that inhibitory efficacy correlates with the presence of GCGGG motifs, with ODN2088 (a prototype G-quadruplex antagonist) demonstrating nanomolar-range TLR-9 antagonism. These pharmacological properties position CpG-ODNs as versatile tools for targeted immune modulation.

Capitalizing on spatial regulation principles, Han *et al*. pioneered a mesoscale nanoplatform for renal-selective delivery of ODN2088 in I/R induced AKI models (**Figure 7**B) 129, 180. This nanoplatform exhibited preferential accumulation in proximal tubular epithelium due to size-dependent renal filtration and charge-mediated endothelial permeation. Administration of this nanoplatform encapsulated ODN2088 at three critical timepoints (-6 h pre-ischemia, 0 h post-reperfusion, +1.5 h post-reperfusion) conferred significant renoprotection, manifesting as 62% reduction in tubular necrosis and 54% suppression of IL-6/IL-1β expression versus free drug controls. Importantly, NP-mediated targeting abrogated systemic TLR-9 blockade in hematopoietic cells, preserving beneficial extrarenal signaling while selectively inhibiting pathologic tubular responses. Pharmacodynamic profiling revealed a 3.8-fold increase in renal ODN2088 bioavailability compared to intravenous administration, validating the nanoplatform's targeting efficiency.

In conclusion, cell type-specific TLR-9 targeting represents a breakthrough in AKI therapeutics. Preclinical data validate this approach as superior to systemic TLR-9 inhibitors, demonstrating preserved host defense capacity alongside enhanced renal cytoprotection. Future translation requires addressing nanoplatform immunogenicity and optimizing dosing schedules for clinical I/R-induced AKI scenarios.

### 3.10 Chemokine receptor type 4 (CXCR4)

CXCR4, a G protein-coupled receptor, and its primary ligand CXCL12, also known as stromal cell-derived factor-1 (SDF-1), play a critical role in stem cell homing, leukocyte migration, and organ development. The interaction between this receptor and its ligand operates through a 'two-step/dual-site' mechanism, initiated by the binding of SDF-1 to the sulfated N-terminus of CXCR4—particularly at tyrosine residue 21 (Tyr21)—which is essential for high-affinity binding and subsequent receptor activation 181.

In the context of kidney pathology, the SDF-1/CXCR4 signaling axis is markedly upregulated following tissue injury. Tögel *et al.* were the first to report the induction of both SDF-1 and CXCR4 expression in renal tubular cells after acute kidney injury 182. This upregulation contributes to increased leukocyte infiltration and actively promotes the development of renal fibrosis. As a result, inhibition of CXCR4 has emerged as a promising therapeutic approach to attenuate renal function decline by modulating inflammatory cell recruitment and suppressing pro-inflammatory responses.

The incorporation of CXCR4 antagonists into nanoplatforms enable targeted therapeutic delivery for kidney diseases. For example, Tang *et al*. developed a siRNA delivery system by modifying chitosan with an α-cyclam-p-toluic acid (CPTA) derivative, yielding a polymer (C-CS) that exhibits dual functionality: CXCR4 targeting and antagonism 130. Similarly, Jogdeo *et al*. conjugated CPTA to inulin to achieve targeted delivery of p53 siRNA 131. In both studies, the resulting nanoparticles exhibited rapid accumulation and prolonged retention in injured kidneys characterized by CXCR4 overexpression. These effects led to efficient gene silencing, reduced cellular apoptosis, diminished inflammatory responses, and improved renal function in models of AKI.

## 4. Conclusions and prospects

The integration of nanotechnology into renal therapeutics has fundamentally transformed pharmacological intervention strategies by enabling precise targeting of pathophysiological processes underlying kidney diseases. Engineered nanoplatforms achieve renal-specific accumulation through optimization of physicochemical properties and ligand-mediated active transport mechanisms, effectively overcoming the limitations of nonspecific biodistribution inherent in conventional therapeutic approaches. Passive and active targeting strategies differ in terms of specificity, design complexity, manufacturing cost, and off-target effects (**Table S2**). Therefore, researchers can select appropriate targeting strategies based on these differences when designing kidney-targeted nanoplatforms.

Despite the promising preclinical outcomes of kidney-targeted nanoplatforms, their translation into clinical practice encounters substantial challenges that warrant rigorous evaluation. A primary concern is the potential for off-target effects. Although active targeting strategies improve renal accumulation, the expression of many target receptors—such as CD44 on immune cells and PSMA in prostate tissue—is not exclusively restricted to the kidneys. This non-specific expression may result in unintended nanoparticle accumulation in non-renal tissues, thereby altering biodistribution profiles and increasing the risk of adverse events. Even passive targeting is constrained by substantial inter- and intra-patient variability among human populations, resulting in less predictable therapeutic efficacy relative to observations in animal models. While biological barriers pose fundamental challenges, significant practical limitations continue to impede the clinical translation of nanomedicine strategies. Scalable manufacturing of complex, multifunctional nanosystems under cGMP standards remains a substantial challenge, particularly with respect to batch-to-batch consistency in physicochemical properties that are critical for therapeutic performance. Furthermore, the predominant reliance on rodent preclinical models contributes to translational gaps, as these models do not fully recapitulate human renal pathophysiology or the natural progression of chronic kidney diseases. Prior to advancing to human trials, comprehensive long-term biodistribution and tissue accumulation studies should be accompanied by systematic evaluation of nanoparticle biodegradation pathways and potential systemic immunogenic effects.

Looking ahead, addressing the key challenges associated with targeting specificity, scalable manufacturing, and long-term safety will require sustained interdisciplinary collaboration. The ultimate goal should shift from merely demonstrating proof-of-concept in rodent models to delivering safe, effective, and clinically translatable nanotherapeutics for patients with kidney diseases.

## Supplementary Material

Supplementary tables.

## Figures and Tables

**Table 1:** Targeting specific components of the nephron and corresponding pathological conditions

	Targeting ligand	Size; charge	Target	Targeting strategy	Targeting strategy	Loaded drug	Disease	Ref.
**PCurNPs**	N/A	~6.5 nm	GBM	Passive	HD < Renal filtration threshold	Curcumin	AKI	73
**GA-NPs**	N/A	~4.5 nm	GBM	Passive	HD < Renal filtration threshold	Gambogic acid	AKI	103
**MSA-QDs**	N/A	~3.7 nm; negative charge	GBM	Passive	Negative charge facilitates the triggering of anionic GBM barriers	N/A	N/A	80
**BPNSs**	N/A	~226 nm; -22.5 mV	TECs	Passive	Flakes structure demonstrates enhanced internalization potential by cells.	N/A	AKI	83
**MNP**	PEG	~386.7nm; +(19.5 ± 0.6) mV	Basement membrane	Passive	Pressure gradient-driven endocytosis	N/A	N/A	95
**TWNDs**	N/A	~3 nm; -35.4 mV	TECs	Passive	HD < Renal filtration threshold	N/A	AKI	104
**Ser-PAMAM**	Ser	~4 nm; +(4.76 ± 0.7) mV	PTCs	Active	Kim-1	N/A	N/A	105
**Ser-PLL**	Ser	~4 nm; +(6.6 ± 3.7) mV	Proximal tubules	Active	Kim-1	^90^Y	RCC	106
**S-G-R**	Ser	63 ± 4 nm; +(3.9 ± 0.3) mV	PTCs	Active	Kim-1	RA	AKI	107
**SC-TK-SS31**	Ser	NA	PTCs	Active	Kim-1	SS31	AKI	108
**Ser-HPEC**	Ser	~40 nm; -(6.7 ± 0.5) mV	PTCs	Active	Kim-1	HPEC	AKI	109
**PCCS**	Ser	~85 nm; weak electro positivity	PTCs	Active	Kim-1	CPPO; Ce6	AKI	110
**Dex/PFP@LIPs-BMS-α**	BMS-α	~190 nm; -(50.5 ± 8.14) mV	GBM	Active	MC1R	Dex	PHN	111
**ESC-HCM-B**	BMS-α	~64 nm; -(18.7 ± 1.5) mV	GBM	Active	MC1R	mTORC1	DN	112
**Cur/Res@FA-F127/TPGS**	FA	101.5 ± 2.3 nm; negative charge	TECs	Active	FR-α	Cur; Res	AKI	113
**BPFe**	BSA	~2.7 nm	PTECs	Active	Megalin receptor	PDA; Fe	AKI	114
**Modified-PEI polyplexes**	Aminoglycoside	~155 nm; +(15.2 - 29.4) mV	PTECs	Active	Megalin receptor	EGFP plasmid DNA	NA	115, 116
**Cel@LU-CA-CS**	CS	~95.0 nm; +(20.0 ± 3.2) mV	PTECs	Active	Megalin receptor	LU; Cel	AKI	117
**Cil/Dex/H-Dot**	Cilastatin	NA	PTECs	Active	Megalin receptor	Dex	AKI	118
**FU-PVU73**	Fucoidan	~240 nm; -52 mV	TECs	Active	P-selectin	PVU73	AKI	119
**OSA-Fucoidan/Cur**	Fucoidan	~100 nm; -(43.7 ± 0.9) mV	TECs	Active	P-selectin	Cur	AKI	120
**T-pBMA**	TUDCA	~250 nm; -30 mV	TECs	Active	P-selectin	pBMA	AKI	121
**HALP**	HA	~122 nm; -(25.7 ± 0.5) mV	TECs	Active	CD44	Cur	AKI	122
**BA-loaded HA-Br**	HA	~84.6 nm; -18.7 mV	TECs	Active	CD44	BA	AKI	123
**HA-MNP-DXM**	HA	~58.8 nm; -(17 ± 2.9) mV	TECs	Active	CD44	Melanin; DXM	AKI	124
**T-Fc-GLU-IVM-NPs.**	GLU	~100 nm; -(20 - 40) mV	PTCs	Active	PSMA	IVM; Q10	AKI	125
**Pc/C5A@EVs**	MSC-EVs	~120 nm; -30 mV	Glomerular endothelial cells	Active	VCAM-1	Pc; C5A	AKI	126
**PC-PLN**	VHPKQHRGGSKGC	~110 nm; +12 mV	Glomerular endothelial cells	Active	VCAM-1	CLT	CKD	127
**MV-DEX**	EVs	~140 nm	Glomerular endothelial cells	Active	VCAM-1	DEX	LPS-induced nephropathy	128
**MNPs containing ODN2088**	ODN2088	~310 nm	PTCs	Active	TLR-9	ODN2088	AKI	129
**C-CS/siRNA**	C-CS	~130 nm; +15 mV	PTCs	Active	CXCR4	sip53	AKI	130
**IC/sip53**	IC	~150 nm; +20 mV	PTCs	Active	CXCR4	sip53	AKI	131

## Abbreviations

AKI: acute kidney injury
GFB: glomerular filtration barriers
GBM: glomerular basement membrane
MSA: mercaptosuccinic acid
ROS: reactive oxygen species
PEG: polyethylene glycol
PLGA: poly(lactic-co-glycolic acid)
KIM-1: kidney injury molecule-1
TIM-1: t-cell immunoglobulin and mucin domain-containing protein 1
PS: phosphatidylserine
RA: rosmarinic acid
SC: ser-modified chitosan
TK: thioketal
MC1R: melanocortin receptor 1
FR-α: folate receptor α
LDL: low-density lipoprotein
EGF: epidermal growth factor
RAP: receptor-associated protein
LMWC: low-molecular-weight chitosan
I/R: ischemia-reperfusion
SCRs: short consensus repeats
TUDCA: taurodeoxycholic acid
HA: hyaluronic acid
PSMA: prostate-specific membrane antigen
VCAM-1: vascular cell adhesion molecule-1
MSC-EVs: mesenchymal stem cell-derived extracellular vesicles
TLR-9: toll-like receptor 9
CXCR4: Chemokine receptor type 4
SDF-1: stromal cell-derived factor-1
CPTA: α-cyclam-p-toluic acid
