# Supplementary material for: Kidney-targeted nanoplatforms: Strategies and applications
Source: Theranostics. 2026 Jan 1;16(6):3011–31. doi: 10.7150/thno.126217 (PMC12775796; doi:10.7150/thno.126217)
Supplement: Supplementary file 1 — Supplementary tables. [file thnov16p3011s1.pdf]

Table S1 Comparison of Active Targeting Strategies for Renal Nanomedicines

Targeting molecule	Key ligand	Expression pattern	Advantages	Limitation
**Kim-1**	L-serine	Highly upregulated in injured proximal tubule epithelial cells after damage.	High specificity for injury sites with promotion of phagocytosis for apoptotic debris clearance.	Negligible expression in healthy tissues may restrict the therapeutic window; ligand conjugation can alter pharmacokinetic profiles.
**MC1R**	BMS-α	Predominantly expressed on podocytes; high density in the renal cortex.	Activation of the cAMP-PKA signaling pathway, leading to anti-inflammatory and antioxidant effects.	Low receptor density in renal cells; targeted delivery systems must overcome the glomerular filtration barrier.
**FR-α**	Folic acid	Highly overexpressed on the apical membrane of proximal tubules.	High internalization efficiency, adaptability for diverse payload delivery, and robust renal targeting capability.	Targeting efficiency may be modulated by renal function; potential competition with endogenous folate limits specificity.
**Megalin**	Albumin, aminoglycosides, chitosan derivatives	Highly expressed on the brush border of PTECs.	High-capacity endocytosis mediation, broad compatibility with therapeutic agents, and significant renal	Susceptibility to interference from competitive ligands; risk of drug accumulation and

			accumulation facilitation.	associated toxicity.
**P-selectin**	Fucoidan, TUDCA	Upregulated on activated endothelial cells and platelets post-I/R injury.	High specificity for inflammatory sites with inhibition of neutrophil infiltration.	Expression in other inflammatory conditions results in limited tissue specificity.
**CD44**	HA	Overexpressed in peritubular areas during ischemic AKI.	Customizable responsiveness to pathological microenvironments, versatility in nanocarrier system integration, and favorable cellular internalization.	Expression on immune cells raises the potential for off-target effects.
**PSMA**	Glutamate-Urea-R analogs	Specifically expressed on the brush border of proximal tubules; absent from glomeruli.	Exceptional kidney specificity with enzymatic activity-driven site-specific drug release.	Structural complexity poses challenges for synthesis; targeting efficacy may be influenced by enzymatic activity.
**VCAM-1**	VHPKQHR peptide, MSC-EVs	Upregulated on glomerular endothelial cells under inflammatory conditions.	Marked inflammation-specific targeting, chronic kidney disease treatment applicability, and efficient endothelial targeting.	Variable expression levels, dependent on the extent of inflammation, contribute to inconsistent targeting

				outcomes.
**TLR-9**	ODN2088	Upregulated in TECs and glomerular cells after injury.	Pathological-physiological state discrimination, combined anti-inflammatory and anti-apoptotic effects.	Dual cell-specific roles (pro-inflammatory vs. protective) necessitate precise spatial and functional control.
**CXCR4**	CPTA, AMD3100, T140/FC131	Overexpressed on injured PTECs.	Dual anti-inflammatory and anti-fibrotic effects, and therapeutic applicability in AKI and fibrotic disorders.	Widespread receptor expression on immune cells may lead to systemic biological effects.

Table S2 Comparison of Passive and Active Targeting Strategies for Renal Nanomedicines

	**Passive targeting**	**Active targeting**
**Core mechanism**	Relies on the physicochemical properties of nanoparticles to passively traverse biological barriers.	Utilizes surface-conjugated targeting ligands to actively bind to overexpressed specific receptors on diseased cells.
**Targeting basis**	Physiological structure of the kidney and disease-induced changes.	Overexpression of specific biomarkers on target cells in disease states.
**Specificity**	Moderate to low.	High.
**Design complexity**	Relatively Low. Primarily involves tuning intrinsic nanoparticle properties.	High. Involves ligand screening, synthesis, and chemical conjugation to nanocarriers, with more complex steps.
**Manufacturing cost**	Relatively Low. Simpler synthesis processes, easier standardization and scale-up.	High. Complex ligand synthesis and conjugation processes, stringent quality control, leading to significantly higher costs.
**Off-target effects**	High. Significant accumulation in RES organs and non-specific distribution within the kidney.	Low. Dependent on ligand selectivity; potential off-target effects if the target is expressed elsewhere.
